# Intensive Longitudinal Data Collection Methods in Health Research: Tutorial for Selecting Measures and Designing a Sampling Protocol

**DOI:** 10.2196/81290

**Published:** 2026-09-08

**Authors:** Jimikaye Courtney, Genevieve Dunton

**Affiliations:** 1 Department of Exercise and Sport Science, College of Arts and Sciences, University of North Carolina at Chapel Hill, 124A Fetzer Hall, Chapel Hill, NC, 27599, United States, 1 9194451520, 1 (919) 962-0489; 2 Department of Preventive Medicine, Keck School of Medicine, University of Southern California, Los Angeles, United States

**Keywords:** intensive longitudinal data, ecological momentary assessment, daily diaries, study design, wearable electronic devices

## Abstract

Intensive longitudinal data (ILD) include frequent and dense repeated measures captured over acute timescales (eg, every second, hour, or day) that are used to investigate within-person processes both within and across days. ILD are collected via wearable sensor data, ecological momentary assessments, or daily diaries and provide unique insights into within-person processes under ecologically valid conditions that can strengthen understanding of temporal relationships among variables and potential causal processes, while also informing the development of just-in-time adaptive interventions. Advancements in mobile and sensor technology have facilitated an explosion of ILD studies that have outpaced formal training in ILD study design. When designing ILD studies, researchers need to make careful decisions about the frequency (ie, how often) and timing (ie, when) of measurements. Decisions about the frequency and timing of measurement are influenced by issues such as variability across individuals and constructs, the purpose of the assessment, concerns about recall biases, saliency, or missing information, and participant needs. The interpretation of study results, causal inferences, and the predictive value of ILD are also impacted by decisions related to the timing of assessments, temporal lags between measures, how a “day” is defined, and data aggregation choices. Due to the increased interest in and adoption of ILD studies, and a lack of formal training, researchers can benefit from guidance on how to design ILD studies. Therefore, this paper aims to provide practical guidance to researchers on how to plan ILD studies using a step-by-step decision-making tutorial with applied health behavior research examples examining phenomena (eg, physical activity and alcohol use) that vary over acute time scales.

## Introduction

Advancements in mobile and sensor technologies have facilitated an explosion of real-time data capture strategies in health behavior research to collect intensive longitudinal data (ILD) of time-varying phenomena [1-5]. ILD include frequent and dense repeated measures captured across acute timescales (eg, every second, hour, or day) [6,7]. ILD are collected via multiple methods, including wearable sensors, ambulatory assessments, experience sampling methods (ESMs), ecological momentary assessments (EMAs), and daily diaries [6,8-11]. These advanced methodologies reflect the increased interest and need for strategies to capture health behavior phenomena that change in intensity or direction across time scales as short as minutes, hours, or days. For example, engagement in health behaviors that impact chronic disease risk, such as unhealthy food intake, alcohol and tobacco use, physical inactivity, and lack of sleep [12,13], varies from day to day. Similarly, psychological and contextual factors that influence health behavior engagement, such as affect, intentions, and social context, can fluctuate rapidly over time. As a result, studies in health behavior and prevention research are increasingly using strategies to capture these types of psychological and contextual factors and behavioral outcomes. Such studies often aim to describe these phenomena, but may also inform the design and implementation of just-in-time adaptive interventions (JITAIs) [14,15].

When using ILD to examine health behavior phenomena that vary across short time scales, researchers often ask questions about temporal dynamics (eg, how variables change over time) to inform intervention and treatment approaches. First, researchers may ask whether these phenomena change as a function of time itself, such as increasing linearly during treatment or demonstrating a curvilinear pattern across the day. Second, researchers may examine within-person effects of time-varying predictors, such as whether greater perceived stress compared with their usual stress level leads to greater consumption of saturated fat in the next few hours. Third, researchers may investigate the extent to which these within-person effects of time-varying predictors change over time or across situations, such as whether a person’s positive affective response during physical activity depends on being alone or with others.

Up until recently, methods to capture the dynamic nature of how these time-varying phenomena change over time were not available. Previously, research on health behavior phenomena typically relied upon cross-sectional, longitudinal, or experimental designs where exposures and outcomes were assessed infrequently (eg, monthly or yearly) across few occasions. These studies used retrospective measures to capture an individual’s usual level of a behavior or indicator such as average daily minutes of physical activity or past month frequency of depressive symptoms. Such approaches are not only vulnerable to recall biases, but they may lack ecological validity because they are not captured in real-world contexts, and they are not conducive to assessing phenomena that vary frequently over time or space or exhibit dynamic patterns of change and fluctuation [16].

Advancements in methods for collecting ILD have overcome many limitations of previous approaches, enabling researchers to capture time-varying phenomena in people’s daily lives. ILD data collection methods, which collect data through wearable sensors, EMAs, and daily diaries, among others, differ from previous methods in how frequently variables are measured and participants’ degree of active engagement with data collection. Wearable sensors can passively collect continuous or semicontinuous ILD every minute or several times per minute about time-varying phenomena such as heart rate, physical activity (eg, acceleration and step counts), location, or alcohol use, and require minimal participant engagement with data collection [17-25]. For example, participants may wear a physical activity sensor while awake, with their engagement limited to removing the sensor while sleeping or charging [21,23,24].

Another common approach to collecting ILD is through ESMs and EMAs (referred to throughout as “EMA”), which require active participant input through multiple self-reports per day of time-varying psychosocial or contextual variables, such as a participant’s emotional states, cognitions, or social or physical contexts [2,4,6,8,10,11,16,26-33]. EMAs can capture time-varying measures before, during, or after specific events, such as during a physical activity bout or while drinking alcohol [26,28]. EMAs may be initiated by researchers (eg, signal- or interval-contingent EMA) or by participants’ behavior (eg, event-contingent EMA), with the goal of capturing time-varying, ecologically valid information about participants’ real-world experiences [4,6,10,11,26,33].

Daily diaries are also an active data collection strategy that includes a single self-report per day of variables, behaviors, or events that are expected to vary from day to day, rather than within a day [6,9,32,34,35]. Daily diaries may ask about a participant’s stress experiences or physical activity or alcohol use behaviors on a given day, as these typically occur only once per day [9,34-36]. Daily diaries may include morning reports of the participant’s previous night’s experiences (eg, sleep quality) or evening reports of the participant’s current day experiences (eg, soda intake) [37,38]. Like EMA, daily diaries also aim to capture participants’ real-world experiences in near real time.

## A Decision-Making Tutorial for Designing a Study Using ILD

These advancements in mobile and sensor technology have outpaced formal training in ILD study design. A recent concept paper partially addressed this need by developing a list of ten topics specific to the design and implementation of experience sampling studies, providing guidance on how to select ESM items, ESM sampling schemes, and practical considerations for real-world implementation of ESM studies [39]. A recently published special issue on ILD designs includes a series of articles addressing ILD considerations related to statistical power, sampling designs, participant experiences, and psychometrics or data quality [40]. The special issue dives deeply into specific features of ILD, providing practical guidance for researchers who are experienced in designing and implementing ILD studies [40]. However, there is a need for an ILD tutorial developed for researchers who are new to or have limited experience with ILD studies, such as graduate students and early career researchers. Additionally, guidance on ILD study design that simultaneously addresses the use of sensors, EMA, and daily diary methods is warranted, as many ILD studies rely on a combination of these methods to answer health behavior research questions. When designing ILD studies, researchers need to make careful decisions about the method used to capture health-behavior phenomena, as well as the frequency (ie, how often) and timing (ie, when) of measurements. These decisions should be based on the a priori research question and consider the characteristics and preferences of the target population. To address these needs for training and guidance, we present a decision-making tutorial for designing ILD studies. Multimedia Appendix 1 provides a detailed, step-by-step guide for navigating the decision-making process.

The tutorial is informed by a synthesis of the authors’ applied experience in designing, implementing, and analyzing ILD studies, their didactic and consulting work with researchers new to ILD study designs, and relevant empirical and methodological literature. Recommendations were developed using an iterative, practice-oriented process that integrates published guidance with common challenges encountered in applied research. The recommendations and examples reflect the authors’ applied research backgrounds, which have shaped the selection of illustrative health behaviors, populations, and design considerations emphasized in the tutorial. As such, the tutorial may not represent the full range of possible ILD applications (eg, clinical or patient populations [41-44]). The tutorial is intended for beginning- to intermediate-level researchers who are interested in examining health behavior phenomena that vary over acute time scales such as minutes, hours, and days. The tutorial provides a step-by-step approach to 10 decisions that need to be made when selecting measures and developing a sampling protocol to collect ILD. The tutorial poses these decisions (in the form of questions) about the nature and timing of the variables and associations involved in the research of interest. Depending on the answers to those questions, the tutorial provides specific study design recommendations that are based on answering the research question of interest. The end of the tutorial provides data processing and analysis recommendations that can facilitate answering the research question based on the study design but should not dictate study design decisions. Although this tutorial and the applied examples emphasize health behavior research, the decisions and methods apply to a wide range of psychological, behavioral, and biological phenomena that may vary dynamically within persons. To facilitate readers’ understanding of how to use the tutorial for investigating health behavior research questions, the design decisions are described with 2 working examples based on the authors’ applied research experiences that represent some of the common research questions about temporal dynamics described above. The dependent variables (DVs) in the examples are health behaviors that vary over acute timescales (minutes, hours, or days) and are commonly investigated in ILD studies – physical activity and alcohol use [45-52]. The independent variables (IVs), momentary affective states, and daily drinking intentions, similarly vary across minutes or days, respectively, and are associated with physical activity and alcohol use behaviors [46-48,50,53-55].

In this study, we propose the following two research questions:

1. In children, are momentary affective states associated with moderate-to-vigorous physical activity (MVPA) levels in subsequent 30 minutes?
2. Do the effects of drinking intentions on same-day drinking behaviors differ based on the experience of daily stressors?

## Decision 1: Determine Whether the IV and DV Change Over Time Within People

The first step is for the researcher to define their primary research question, which will serve as the basis for subsequent design decisions (question 1 in Multimedia Appendix 1). There may be several research questions within a single study, and each question may require different measurement or sampling protocol considerations; therefore, for simplicity, the researcher should start with a primary research question for which decisions about measures and a sampling protocol are made. For secondary research questions, the researcher can repeat the steps in the tutorial and attempt to integrate the design recommendations when possible. Once the primary research question is articulated, the researcher should define their IV and DV with as much specificity as possible (questions 2‐3). Then, the researcher should determine whether the IV and DV change over time within people (eg, whether these are time-varying variables that change within people). If either the IV or DV change over time within people, then the research question may be suitable for an ILD design, and the researcher may proceed with the next part of the tutorial.

Application to research examples: For the first research example, both affective states (DV) and MVPA (IV) change over time within people. Similarly, for the second research example, drinking intentions (IV), daily stressors (moderator), and drinking behaviors (DV) change over time within people, making both research questions suitable for an ILD design.

## Decision 2: Identify the Timescale of Within-Person Changes

The second decision is to identify the timescale of within-person change for the primary DV (question 4), which may be short, such as minute-to-minute changes in heart rate [56], or long, such as changes in disease risk factors (eg, hemoglobin A1_c_) across months or years [57]. ILD studies are most appropriate for examining IVs and DVs that change across minutes, hours, or days. This is reflected by the frequency of ILD measures, with sensors measuring variables several times per minute or hour (eg, heart rate, blood glucose, or bodily movement), EMAs measuring variables several times per day (eg, affect, cognition, or tobacco use), and daily diaries measuring variables once each day (eg, intentions or alcohol use) [58,59]. If the IV and/or DV are expected to show within-person change over timescales of a day or shorter, then an ILD design is likely an appropriate choice. If the IV and/or DV changes over a longer timescale (such as monthly), an ILD study may result in collecting data more frequently or intensely than necessary, which may be costly for researchers and unnecessarily burdensome for participants. Some researchers may choose to use an ILD study design when the DV changes over longer timescales (eg, weeks and months) if they are interested in examining within-person change or variability in the IV as a predictor. However, this type of ILD study design requires slightly different design considerations and statistical methods than traditional ILD studies focused on within-person change in the DV. Therefore, discussion of this type of ILD study is beyond the scope of this paper. For IVs or DVs expected to show within-person change from day to day, a daily diary (ie, once a day) design may be appropriate. For IVs or DVs expected to change over a few hours within a day, wearable sensors or EMA may be appropriate. Finally, for IVs or DVs expected to change over minutes or seconds, wearable sensors may be the best measurement method. If the timescale of within-person change is unknown, it may be more efficient to start with EMAs rather than daily diaries, because data can always be aggregated up to a longer timescale from the EMA measures (eg, day-to-day). In contrast, data cannot be broken down into timescales shorter than the original measure, meaning that starting with daily diaries may be less efficient for identifying the timescale of change when it is unknown.

Application to research examples: For the first research example, investigating associations between momentary affective states and MVPA in the subsequent 30 minutes in children, affective states (ie, a subjective feeling state that a person experiences at a given moment, including both how positive or negative the feeling is [“affective valence”] and how energized the person feels [“affective arousal”]) change within-person over minutes or hours—suggesting that either wearable sensors (eg, affect derived from galvanic skin response or heart rate variability) or EMA (eg, self-reported affect) may be appropriate design choices. MVPA changes over minutes, suggesting that wearable sensors are an appropriate design choice. For the second research example, investigating whether daily stressors moderate the effects of drinking intentions on behaviors, daily stressors (moderator) change from day to day, and daily drinking intentions (IV) may change from day to day—suggesting that daily diaries may be appropriate to measure both variables [47,60-63]. Drinking behaviors (DV) change across minutes or hours, suggesting that sensors or EMAs may be appropriate. In the next design decision, we will select the specific ILD measurement approach to use.

## Decision 3: Determine Whether There Are Valid and Reliable Sensor-Based, EMA, or Daily Diary Measures

The next decision is whether reliable and valid sensor, EMA, or daily diary measures exist to assess the IV and DV of interest. Researchers should first consider whether sensor measures (ie, portable or wearable devices that use mechanical, electronic, or digital hardware and software) are available (question 5). Sensor measures can passively collect objective data (eg, heart rate, skin temperature, blood sugar, blood alcohol, body movement, sun exposure, or geographic location) [17,19,21,22,25] and may generate less participant burden [64] because they do not require manual user input.

If valid and reliable sensor measures are available, then researchers should consider the extent to which using the sensor will result in participant reactivity (ie, when awareness of being measured leads to changes in phenomena of interest; Table 1; question 5a) [65]. To manage participant reactivity, researchers could reduce participant access to information or data generated by the sensor (eg, do not provide access to the accompanying smartphone app, put tape over the sensor’s display).

**Table 1.** Key concepts explained.

Concept	Explanation
Within-person change over time	How much an individual person in a sample or population tends to change over a specific timescale (eg, hours, days, weeks) regarding a given behavior or value of a construct. In the case of ILD^a^ studies, within-person change is typically expected to occur within minutes, hours, or days, making the construct appropriate to study via ILD.
Passive assessment	ILD assessments that consist of collecting continuous or semicontinuous measures of body movements, physiology, location, device use, etc, without active participant engagement, that are often considered “objective” measures and are collected via wearable sensors or smartphones.
Active assessment	ILD assessments that consist of collecting participant self-reported measures of psychological constructs (eg, stress, affect), cognitive constructs (eg, thought processes), or context (eg, social, environmental), that are often considered “subjective” measures and are collected via surveys completed via smartphones or computers once or multiple times per day.
Participant reactivity	When a participant’s behavior or subjective responses alter because they know they’re being studied. Also referred to as a measurement or assessment effect, participant reactivity can also occur because responding to assessments provides a form of self-monitoring or feedback that changes participants' subjective responses, such that their responses are more reflective of their reaction to the assessment rather than to true naturalistic experiences or changes.
Expectancy effects	A form of participant reactivity in which a participant anticipates an upcoming assessment and changes their behavior or context to provide the expected response or outcome, to appear more favorable to researchers or to provide researchers with an “ideal” response, or to improve their ability to respond to the assessment.
Participant burden	When the effort required by the participant exceeds their abilities or motivation, impacting participant compliance and data quality. In ILD studies, wearable sensor-related burden may be related to the requirement to remove or replace, charge, or sync a device. EMA^b^ and daily diary-related burden may be due to the length of the study protocol, frequency or timing of assessments, length of assessments, or length or complexity of individual items.
Naturalistic processes	Processes, such as behaviors, experiences, or cognitions that occur in people’s real-world environments and that would occur naturally whether or not the research study is taking place. ILD studies often attempt to collect data in people’s real-world contexts to maximize the ecological validity of findings.
Recall bias	Type of response bias in which a participant fails to accurately remember a past event or experience or to leave out relevant details when reporting them. In ILD, recall bias is influenced by design features, such as the timeframe across which participants are asked to recall data in EMAs (eg, “currently,” “past 10 min”) or daily diaries (eg, “today,” “since waking”)
Availability heuristic	A type of mental shortcut participants use when responding to questions, in which they rely on immediate or easily accessible information to inform their responses, which can be influenced by the timeframe for recall or item wording in ILD studies.
Saliency bias	A type of response bias in which more personally relevant or more intense experiences are more likely to be recalled, which is related to the availability heuristic. In ILD studies, daily diary items may be particularly impacted by saliency biases because participants are asked to recall constructs across the entire day, such as stressful events.
Peak effect	A type of response bias in which the most intense experience (ie, “peak”) disproportionately influences a participant’s response. For example, an extreme moment of anger unduly influencing the report of negative affect.
End effect	A type of response bias in which the most recent experience in time (ie, “end”) disproportionately influences a participant’s response. For example, the last social interaction of the day influencing the participant’s report of social connectedness.
Sampling window	The timeframe a researcher is interested in investigating. In ILD studies, this may be the entire 24-hour period, waking hours, sleeping hours, or a discrete event, such as a physical activity bout or a drinking event.
Participant schedules	The typical timing of daily behaviors being investigated among participants, such as sleep and wake times, class times, or work start and stop times. In ILD, participant schedules may be similar or different between participants and may be consistent or inconsistent from day to day within participants, which influences how sampling windows are specified for a study.
Between-person differences	When the association between the IV^c^ and DV^d^ reflects a difference between individuals in a sample. For example, when examining the association between stress and physical activity, a between-person effect could be that people who experience higher stress on average compared to other people are less physically active.
Within-person differences	When the association between the IV and DV reflects a difference between moments or days in an individual participant. For example, when examining the association between stress and physical activity, a within-person effect could be when a person experiences more stress than usual when they are more physically active than their average amount.
Concurrent effects	These are associations in which the IV and DV are expected to cooccur in time, also referred to as synchronicity. For example, it may be hypothesized that a mother’s stress level will concurrently impact their child’s stress level.
Lagged effects	These are associations in which the IV is expected to impact a DV that occurs at a later point in time, also referred to as sequentiality. For example, it may be hypothesized that a mother’s stress level will impact her parenting behavior later in the day, which may in turn influence a child’s subsequent behaviors.
Observation	The lowest level “unit” of analysis that will be used in hypothesis testing, statistical analyses, or the interpretation of results. This may be the momentary assessment, a discrete occasion or event, a given timeframe (eg, every 2 h), or the entire day.
24-hour day or Calendar day	A traditional 24-hour day based on a calendar date that begins at midnight and ends at 11:59 PM.
Study-centric day	A 24-hour cycle that is based on a study design feature or phenomenon of interest. For example, a researcher interested in studying a person’s alcohol use behaviors may choose to align the day start with a 9 AM morning assessment about past day drinking and end the day at 8:59 AM on day n+1 to ensure that all drinking behaviors are consequences are attributed to the correct functional day.
Person-centric day	A ”day” that is based on an individual’s sleep-wake cycle and does not follow a 24-hour cycle, with each day differing between and/or within participants. This may include entire days or person-centric waking or sleeping periods. For example, each person-centric day may start when they wake up on day n and end when they wake up on day n+1, such that some ”days” may be longer or shorter than 24 hours.
Measurement burst design	A type of intensive longitudinal design consisting of repeated clusters (“bursts”) of high-frequency assessments separated by intervals in between the bursts without any assessment occurring. This design allows researchers to capture short-term dynamics within bursts while also examining longer-term change between bursts. It is particularly useful when the DV changes over longer timescales, when within-person variability in a time-varying variable changes over longer timescales, when associations between time-varying variables vary over longer time scales, when key variables reflect relatively rare or time-bound events (eg, weekend alcohol use or phases of the menstrual cycle), or when it is necessary to balance temporal resolution with participant burden.

a ILD: intensive longitudinal data.

b EMA: ecological momentary assessments.

c IV: independent variable.

d DV: dependent variable.

Next, researchers should ask whether the sensor is cost-effective and whether there are resources (including personnel and software) to support using it (question 5b-c). Some sensors cost hundreds of dollars, which can be prohibitive for studies with smaller budgets or those requiring large samples. Also, sensors may require substantial research personnel resources to provide instructions on use and troubleshoot technical problems [66]. They may also require technological expertise to program, fix, and process data [22,66].

Finally, researchers should consider participant burden imposed by the sensor. Participant burden occurs when the effort (time, physical, and attention) required exceeds the ability or motivation to engage with or use a measure, which impacts data quantity and quality (question 5d) [67]. When burden from a sensor is higher than participants can handle, researchers may choose to use a less burdensome self-report measure instead, such as EMA or a daily diary, or they may consider a design with less frequent assessments (eg, every other week). Characteristics of the target population are also related to participant burden. For example, people who are less comfortable with technology may find EMAs more burdensome than tech-savvy populations [68], although well-designed study protocol, participant training, and user-friendly smartphone apps can address issues of participant burden and enhance compliance [52,68,69].

Some constructs may be too subjective in nature (eg, perceptions, motivations, intentions, and cognitions) to be assessed through a sensor, requiring self-reports via EMAs or daily diaries instead. In this case, researchers should determine whether there are acceptable or widely used items and/or instruments to assess those constructs (question 6). Ideally, researchers will select measures that were developed and validated using within-person longitudinal data, as measures developed from between-person cross-sectional data may not have the same factor structure at the within-person level, making it unclear whether shifts in variables reflect true within-person changes or measurement error [70-73]. While within-person validated measures are not yet available for all constructs, in recent years, freely available repositories have emerged where researchers can share EMA tools, measures, and reliability and validity information such as the Experience Sampling Method item repository [74]. If a within-person validated measure is not available, researchers may adapt an existing between-person cross-sectional measure (using appropriate recall time frames), while acknowledging that the measure was not validated for within-person use. If feasible, researchers could simultaneously conduct a psychometric study evaluating the reliability, validity, internal consistency, and/or factor structure of the items adapted from the between-person instrument (eg, using multilevel factor analysis to explore within- and between-person measurement model differences [72,73,75]), contributing to the ongoing need for psychometrically sound EMA measures [70,71,76]. An important point to consider is whether variability captured by EMA or daily diary measures represents true changes in an underlying construct or instead is indicative of poor reliability of the measure [70,71,76]. In contrast, it is necessary to understand whether lack of variability in EMA or daily diary measures is an artifact of careless responding (ie, participant not using the full range of response options due to lack of interest or fatigue) or true stability in a construct. It is possible to investigate these concerns by determining whether changes in EMA and daily diary measures correspond to changes in monitor-based or biologically-based indicators captured across the same timeframe (eg, accelerometry, cortisol, heart rate, and galvanic skin response) [77]. Other strategies to limit measurement error are to use a larger number of items to assess a construct, ensure adequate sampling frequency, avoid unreliable measures, and use appropriate statistical analytic strategies (eg, multilevel modeling or latent variable modeling) to differentiate measurement error from true variability [78].

If researchers find an acceptable EMA measure, they should consider whether answering EMAs will result in participant reactivity to a sufficient degree that it could alter study outcomes (question 6a).

Researchers should also consider whether actively responding to a real-time EMA prompt interrupts a naturalistic process that a participant is experiencing such as a romantic encounter, conversation, physical activity session, or emotional moment (question 6b). If so, researchers could consider measuring the construct passively using a sensor or retrospectively via an end-of-day daily diary [34]. Researchers should additionally ask whether the naturalistic process impacts the participant’s ability to provide valid and/or reliable EMA responses (eg, being intoxicated while responding to questions about drinking alcohol; question 6c). Finally, similar to using a sensor, researchers should ask whether the study has the financial, personnel, and informational resources to support implementing EMAs. If not, a sensor or daily diary measures could be considered (question 6d).

Finally, if researchers are contemplating using daily diary measures, they should seek out reliable and valid items and instruments to use (question 7). In some cases, items originally designed for EMA can be easily adapted for a daily diary by adding, “Over the past day.” However, in some circumstances, memory errors and biases including peak and end effects can be introduced by asking participants to retrospectively report constructs up to 24 hours later [79]. If reliable and valid daily diary instruments are available, then researchers should consider whether these measures are likely to be impacted by expectancy effects, which occur when a participant has knowledge that a survey is about to occur and changes their mood, location, or behavior in preparation for the survey.

Regardless of the method selected for an ILD study (ie, wearable sensor, EMA, or daily diary), researchers should carefully consider privacy and data security implications. Many commercially available smartphone apps and wearable sensors require participants to agree to user terms and privacy policies that are outside of researchers’ control, which may allow data sharing with developers or third-party entities [80]. This can introduce risks related to confidentiality, data ownership, and secondary data use [80,81]. Additionally, mobile sensing and EMA approaches often involve the collection of highly sensitive, potentially identifiable data (eg, location or health information), increasing the risk of unintended disclosure or reidentification. Participants may also also be required to accept app-specific data use agreements, which the researcher has no control over [80-82]. Researchers should clearly communicate data access rights and potential confidentiality risks during the informed consent process [81].

Application to research examples: When studying associations between affective states and MVPA in the subsequent 30 minutes in children, wearable sensors are ideal to measure MVPA due to the timescale of change (seconds or minutes), the time frame under investigation (30 min), and minimal concern about participant burden and reactivity to sensors. The population of interest (children) also warrants the decision to use wearable sensors, as self-reports of past 30-minute MVPA via EMAs may be too cognitively burdensome for children to complete—resulting in unreliable data. To measure affective states, EMAs were selected due to the timescale of change (hours), minimal concern about participant reactivity and expectancy effects, and acceptable participant burden, and having resources to support personnel and software costs.

When investigating daily stressors moderating the effects of drinking intentions on drinking behaviors in adults, alcohol use can be measured via sensors, EMAs, or daily diaries. However, EMAs are subject to expectancy effects and biases due to alcohol consumption, and daily diaries are subject to recall biases and miss key features of drinking that often occur while people are sleeping (ie, descending limb of drinking or alcohol elimination). Therefore, passive wearable sensor measures were deemed most appropriate. In contrast, there are reliable and valid daily diary measures of stressful experiences that are not subject to expectancy effects and capture psychological stress experiences and responses, leading to the selection of daily diaries to measure stressors. Similarly, although drinking intentions could vary within a day, the research question was specifically investigating daily intentions. Additionally, drinking intentions are subjective, warranting the use of daily diaries.

## Decision 4: Determine Timing and Frequency of Sampling (EMA Studies Only**)**

The next decision when using EMAs is determining when and how frequently to collect data. If asking participants about their “current” state or “the past 10 minutes,” recall biases will be minimal. However, as the recall time frame increases, participants are more likely to use mental shortcuts to inform their responses, such as relying on the most easily accessible information (ie, availability heuristic) or being influenced by personally relevant or intense experiences (ie, saliency bias, peak and end effects) [83]. The wording of EMA items may also change participants’ recall, how they interpret questions, or how they respond [83]. Researchers should aim to select an EMA sampling schedule that minimizes these types of biases. To do so, they need to identify the time frame across which participants can reasonably recall the construct, when the construct occurs throughout the day, whether it is susceptible to expectancy effects, and whether it occurs during specific events or in specific contexts. For example, if the construct occurs at the same time each day (question 8), such as a regular morning physical activity bout, or if it has low susceptibility to expectancy effects, then an interval-contingent design (ie, same time each day) with EMAs close to when the construct occurs may be appropriate. An interval-contingent design is also appropriate when a researcher is interested in measuring the level of a construct at the same time each day (question 8a). If the construct occurs at different times each day, such as a stressor, or if it has higher susceptibility to expectancy effects (question 9) [3,84], then a signal-contingent design (ie, random times throughout each day) is likely a better choice. Finally, if researchers are interested in measuring the construct during a specific event or behavior (question 10), such as measuring affect during a drinking event, then an event-contingent design is most appropriate. Selecting a particular type of EMA sampling schedule in turn influences EMA item wording, recall windows and, for signal-contingent designs, the frequency of EMA measures.

When designing an EMA sampling protocol, researchers should determine the frequency and length of the recall period. If using a signal-contingent design, then researchers should consider how frequently the construct changes across the day (question 11). Depending on the frequency (eg, every 2‐4 h), researchers may consider sampling 2 to 16+ times per day for 7 to 14 days, for example. However, if more frequent sampling is necessary to capture natural variation in the construct (eg, every hour), then researchers may need to consider a shorter study period (eg, 2‐4 days) to minimize burden and maximize data quality [85]. For example, in the TIME (Temporal Influences on Movement and Exercise) study, a signal-contingent EMA protocol prompted once during every waking hour of the day for 4 days during each bimonthly measurement burst [86].

Researchers should also consider how long after a behavior or experience a participant can reliably recall the phenomena (question 12). If the construct becomes more vulnerable to recall biases over elapsed time (such as intensity of affective valence), the length of the EMA recall window should be short (eg, “Right now, how are you feeling?” or “In the past 15 min, how have you been feeling?”) [48]. In contrast, some constructs such as concrete behavior, social encounters, or locations visited may be reliably reported across longer EMA recall windows (eg, “In the past 4 hours, have you spent any time with your child?”) [87,88]. The ideal recall window may differ between participants. For example, children may find it more difficult than adults to report feelings across the past 15 minutes. It is important to note, however, that the EMA recall window should not be longer in duration than the spacing between adjacent EMA measures of the same construct to avoid overlapping or double assessments. For example, if EMA surveys are prompted every 2 hours, then the recall window should not be longer than 2 hours.

**Figure 1.**
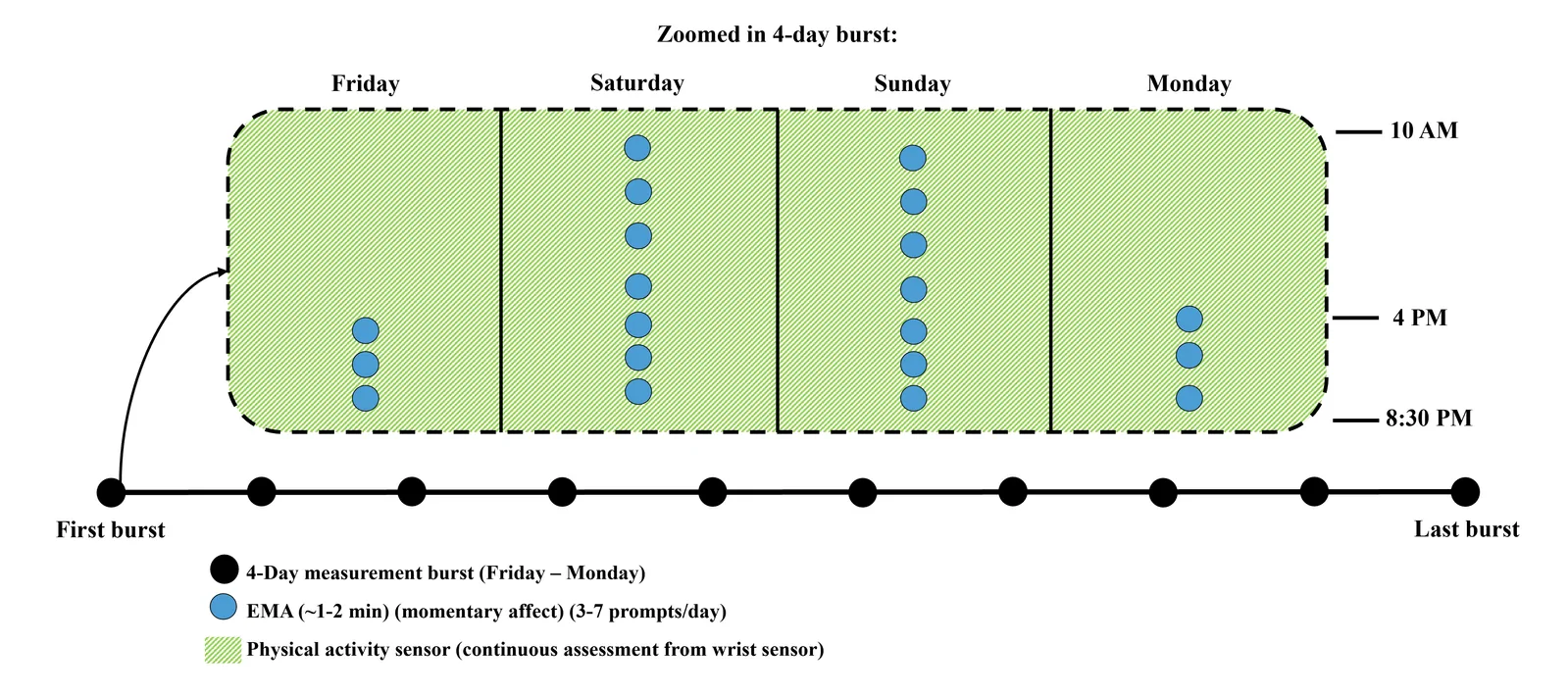
Decision 4: Example intensive longitudinal data protocol using a hybrid interval-signal-contingent ecological momentary assessment design with continuous sensor measures of physical activity via multiple assessment bursts with intensive sampling across four days to balance reliability and power while minimizing participant burden. Random prompts sent between 4 PM-8:30 PM on Friday and Monday and 10AM -8:30 PM on Saturday and Sunday. EMA: ecological momentary assessments; ILD: intensive longitudinal data.

Application to research examples: For investigating affective feeling states in children, we selected EMAs as the most appropriate measure. The EMA sampling design needed to minimize burden on children and account for times of day when they cannot or should not receive prompts. Therefore, a hybrid design with interval and signal-contingent EMAs was selected (Figure 1). During each 2-hour window (interval-contingent component), an EMA survey was prompted at a random time (signal-contingent component). The recall time frame was momentary, asking children about their affect at the given moment (eg, “How happy do you feel right now?”), to avoid issues of recall bias, such as peak and end effects that could occur if asking children about their daily affect at the end of the day.

## Decision 5: Select Sampling Windows and Time(s) of Day When Assessments Occur (Sensor and EMA Studies Only)

**Figure 2.**
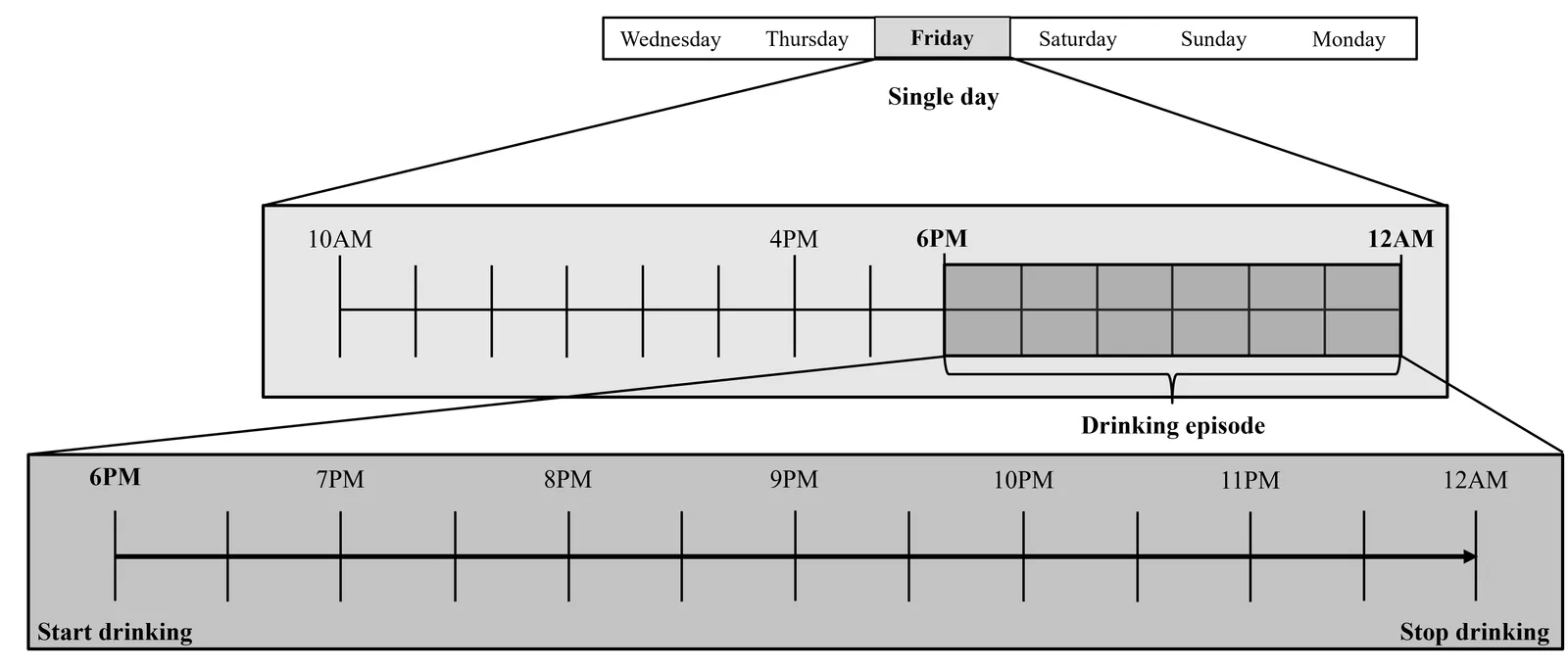
Decision 4: Example Intensive longitudinal data (ILD) protocol using event-contingent ecological momentary assessments (EMAs) during drinking episodes (Courtney and Russell [47]).

The next decisions involve selecting the sampling windows and time(s) of day that assessments should occur (question 13). For example, a researcher may only be interested in social interactions when the participant is at work or screen time in 2 hours leading up to bedtime. In these situations, collecting data outside of these sampling windows may be unnecessarily costly and burdensome. If a researcher is interested in a specific event, such as a high-intensity drinking episode, then they should use an event-contingent design with participants completing EMAs and wearing an alcohol sensor during the drinking episode [46,49] (Figure 2). Additionally, if the research question is about eating behaviors, the researcher may choose to use an interval-contingent design that triggers EMAs 3 times per day immediately after known mealtimes.

Next, researchers need to address whether to use the same EMA sampling window for all participants and/or for each day of the week. If participants’ sleep-wake schedules differ, such as for shift versus nonshift workers [89], then each participant may need a personalized EMA daily sampling window [87]. Researchers also need to determine whether a given participant or all participants have a similar sleep-wake schedule for each day of the week (question 15). If participants have very different schedules, such as going to sleep later and waking up later on weekend days, then sampling windows should be tailored for each day of the week or weekends versus weekdays. Real-time customization of prompting schedules is also possible, such that each day participants are asked when they anticipate going to sleep that night and waking up the next day [86]. Customized schedules can increase compliance by reducing burden and unpleasant sleep interruptions; however, they may be impractical, too costly to program, or create data processing challenges. If customization is too costly or burdensome, then the researchers may want to consider screening out participants with unusual sleep-wake schedules (eg, screen out shift workers), using a sampling schedule that does not include early morning or late evening assessments, or using only event-contingent EMAs.

When making design decisions about sampling windows and schedules, considering the needs and preferences of the target population can help reduce participant burden and missingness. In some cases, surveying members of the target population before making design decisions can help researchers identify ideal sampling windows or the need for tailored or real-time customized schedules. For example, the ideal time of day for asking participants to complete morning diaries of past-day behaviors is likely to differ between college students versus older adult populations. College students may require a later sampling window time (eg, 10 AM-2 PM) whereas the older adults may require an early sampling window time (eg, 7 AM-11 AM).

Application to research examples: For studying affect and subsequent 30-minute MVPA in children, EMAs were triggered on weekday afternoons and evenings and throughout the day on weekend days to avoid interrupting school and sleep time. Using the same schedule across all participants was appropriate given that school schedules are fixed (eg, classes end at 3 PM each day) and the sampling windows were narrow enough to avoid interrupting sleep time, thereby not requiring customized windows.

For investigating daily stressors and drinking intentions and behaviors, there were 2 daily diaries per day, with the sampling windows selected to go from 9 AM-11 AM and 8 PM-10 PM across all weekdays and weekend days. The 9 AM schedule for the morning diary ensured that intentions were reported before drinking behavior, as participants are unlikely to drink that early in the morning. The 8 PM schedule for the evening diary of stressful events avoided interrupting sleep time while reducing recall biases about the day’s stressors. Due to the nature of alcohol metabolism, with alcohol elimination occurring for many hours, often while individuals are sleeping, the decision was made for participants to wear the sensor 24 hours/day. Similarly, inter- and intraindividual variability in timing of alcohol use across weekends and weekdays also warranted the use of continuous sensor wear. As the timing of daily diaries were selected with consideration of late-rising and early bedtimes, and since diaries were available for completion for 2 hours after each prompt, the decision was to use the same schedule for all participants.

## Decision 6: Delineating Temporality of the Associations

The next decision involves researchers specifying the nature of temporal associations that they are hypothesizing, which is a useful step toward better understanding potential causal processes. Whether the associations are thought to be primarily within- or between-persons and concurrent or lagged in time have design implications in terms of statistical power and the sampling timing. Researchers should first determine what type of association they are interested in testing (question 16). If they are examining whether within-person changes in the IV (eg, positive affect assessed every 2 h through EMA) are associated with within-person changes in the DV (eg, MVPA min in 30-min window after the EMA), then the study should include enough observations nested within enough people to detect within-person effects [90,91].

**Figure 3.**
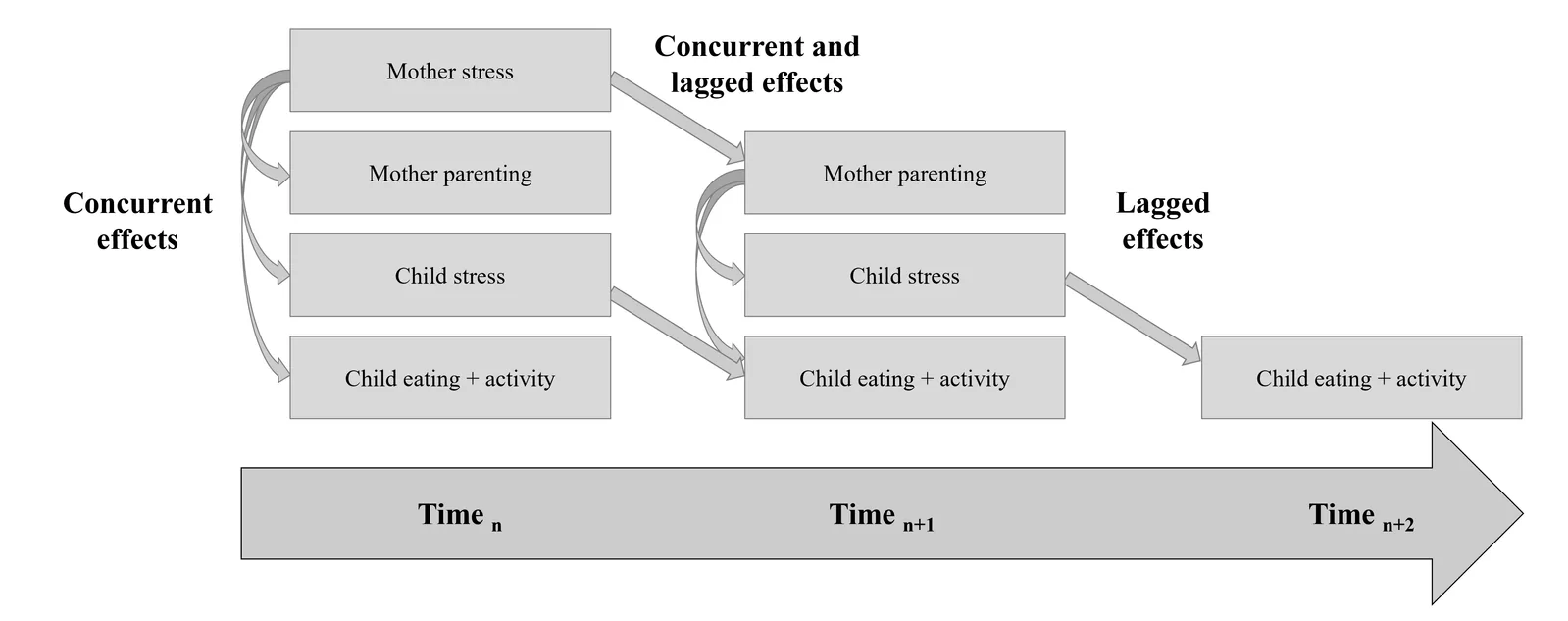
Decision 6: Example of concurrent and lagged effects for examining the causal associations (Redrawn from Dunton et al [87]).

If a researcher is examining whether within-person changes in the IV are associated with within-person changes in the DV, then researchers should first consider whether the IV is hypothesized to be associated with the DV at the same point in time (ie, cooccurring effect) or a later point in time (ie, lagged effect; question 17). For the cooccurring effect, the design would need to ensure that affect and physical activity are assessed concurrently (eg, participants respond to EMA while doing physical activity) whereas the latter hypotheses would require a design that captured physical activity after each EMA prompt and linking data through date-time stamps (Figure 3).

Second, if the researcher hypothesizes that the IV is associated with the DV at the same point in time (ie, cooccurring effect), then they need to specify the “duration” of that point in time (question 18). For example, one could hypothesize they are associated with each other during the same 1-hour period (eg, types of social interactions over the past hour are associated with level of positive affect over the past hour). If using EMA, the item wording would need to specify the duration of the recall period (ie, “In the past hour…”).

Third, if the researcher hypothesizes that the IV is associated with a DV occurring at a later point in time (ie, lagged effect), then it is necessary to specify how much later (eg, in minutes, hours, or days; question 19). For example, if a researcher is interested in understanding whether perceived stress at any point of the day (IV) is associated with asthma symptoms occurring over the next 4 hours (DV), then the sampling frequency and item wording need to reflect these temporal associations [30].

Application to research examples: The first research example explicitly asks whether momentary affect is associated with MVPA in the subsequent 30 minutes. This requires linking sensor data to EMA survey responses and then calculating time spent in MVPA during the subsequent 30 minutes (leading observations). In the second research example, the question is investigating within-person, daily associations between intentions and drinking behaviors, with daily stress as a moderator. The design includes morning diaries about drinking intentions and evening diaries about daily stressors, including reports of the time of day the stressor occurred. This type of design requires linking morning and evening diaries by study day and linking sensor data to the diary data. To ensure temporal precedence, time stamps for when morning diaries were completed and for when stressors occurred must be linked to the sensor data so that (1) only drinking data which occurs after the morning assessment of intentions are included and (2) only stressors that occurred after the morning assessment of intentions and before drinking onset are included. Multimedia Appendix 2 provides example R code using timestamps from the morning diary and stressor occurrences to establish temporal precedence, followed by removing all stressors that occurred before the morning diary.

## Decision 7: Select Number and Type of Days to Assess

Once the temporal and causal associations are specified, the next decision is determining the number and types of days across which the ILD sampling protocol should be implemented. The key challenge is balancing the need for adequate representation across time and statistical power (more observations are typically better) with the potential response burden (fewer observations are typically better). If the research question concerns between-person differences and conceptualizes either the target IV or DV at person level (ie, their usual level of a behavior), researchers should consider how many days will provide adequate representation or between-person reliability (ie, internal consistency) for the target IV and DV (question 20), with reliability meaning the extent to which any given set of assessments approximates the typical level of the construct [92]. For constructs that are generally stable for a person across time, such as the time of the day that someone starts work, only a few days may be needed. However, for constructs that fluctuate from day to day, such as dietary intake, many more days may be needed to obtain adequate representation of one’s typical daily nutrient intake [93]. A different but associated concept is within-person (ie, idiographic) reliability, which addresses how reliably one can detect fluctuations within an individual over time (ie, time-varying states) beyond measurement error as described in decision 3 [78].

Related to the total number of days are questions about whether weekend and/or weekdays are needed (question 21) and the total number of observations per person (question 22). Some questions, such as those pertaining to children’s social interactions at school, may only require weekday assessment whereas other questions, such as those related to overall sleep patterns, would require weeknight and weekend night assessments. Which days to sample for a given phenomenon may also differ depending on the target population. For example, among college students, drinking is more likely to occur on Thursday-Saturday, the “social weekend”; therefore, researchers studying college student drinking may only need to sample those days. However, if studying alcohol use in other adult populations or individuals with an alcohol use disorder, it may be appropriate to sample all days of the week. Finally, if a researcher is using EMA as the primary measurement modality with multiple assessments per day, then they need to consider how many observations per person are needed to ensure adequate statistical power. It is beyond the scope of this tutorial to provide in-depth guidance for power analyses in ILD studies; however, we will briefly state that multiple approaches can be used for estimating power in ILD (eg, simulation or summary-statistics based approaches) and, as in all power analyses, the sample size and number of observations needed depend on the primary effect researchers aim to detect (eg, within-person, between-person effects, and cross-level interactions) [94-97]. Due to the multilevel nature of ILD, power analyses should also account for the estimated intraclass correlation coefficient, which reflects the proportion of variance attributable to between-person differences and influences the amount of independent information contributed by repeated observations [94-97]. For example, a Monte Carlo simulation study found that, with 70 observations per person, a sample size of 15 participants yields 99% power to detect within-person (level 1) effects [94]. More participants were needed to detect between-person (level 2) effects; specifically, 30 participants with 70 observations yield over 80% power to detect between-person effects [94]. Given the complexity of power analyses for ILD, we recommend that readers consult a biostatistician and refer them to the PowerAnalysisIL Shiny app (Ginette Lafit) on GitHub to aid their research team in conducting power analyses for their ILD studies [94].

The next questions relate to balancing reliability and power requirements while minimizing participant burden. When participants experience greater burden due to the behavioral or cognitive effort involved in responding, the quality and/or quantity of data can be reduced [98,99]. If the number of days or assessments per day appear to be too burdensome (questions 23 and 24), then the researcher could consider altering the study design such as having assessments occur on random days with no-assessment days in between (eg, one day on followed by one day off) or multiple assessment bursts with a short string of continuous days (eg, 5 d) separated by multiday breaks (eg, 5 d; Table 1).

Finally, researchers should consider whether they expect the strength of within-person associations to change or remain stable over time (question 25). If they anticipate that a within-person association (eg, whether stress leads to increased hunger in the next hour) remains stable across weeks, months, and years, then they may choose to use a single measurement burst design (eg, a single period of continuous days). However, if they expect that the within-person association changes in strength over longer periods of time (eg, due to exposure to an intervention or historical events), then a multiple measurement burst design with bouts of continuous assessment days separated by the time interval of expected change (eg, weeks, months, or years) should be considered.

Application to research examples: For investigating affect and MVPA in children, the decision to only assess Fridays-Mondays necessitated a measurement burst design to collect sufficient observations. For example, 10 measurement bursts, separated by at least 14 no-assessment days, with 4 days of data collection during each burst permits collecting sufficient observations without undue burden (Figure 1). For investigating daily drinking intentions, stress, and behaviors, at least 30 sequential days of observation are warranted, as the research question is about day-level associations in adults.

## Decision 8: Define What a “Day” Means in the Study

**Figure 4.**
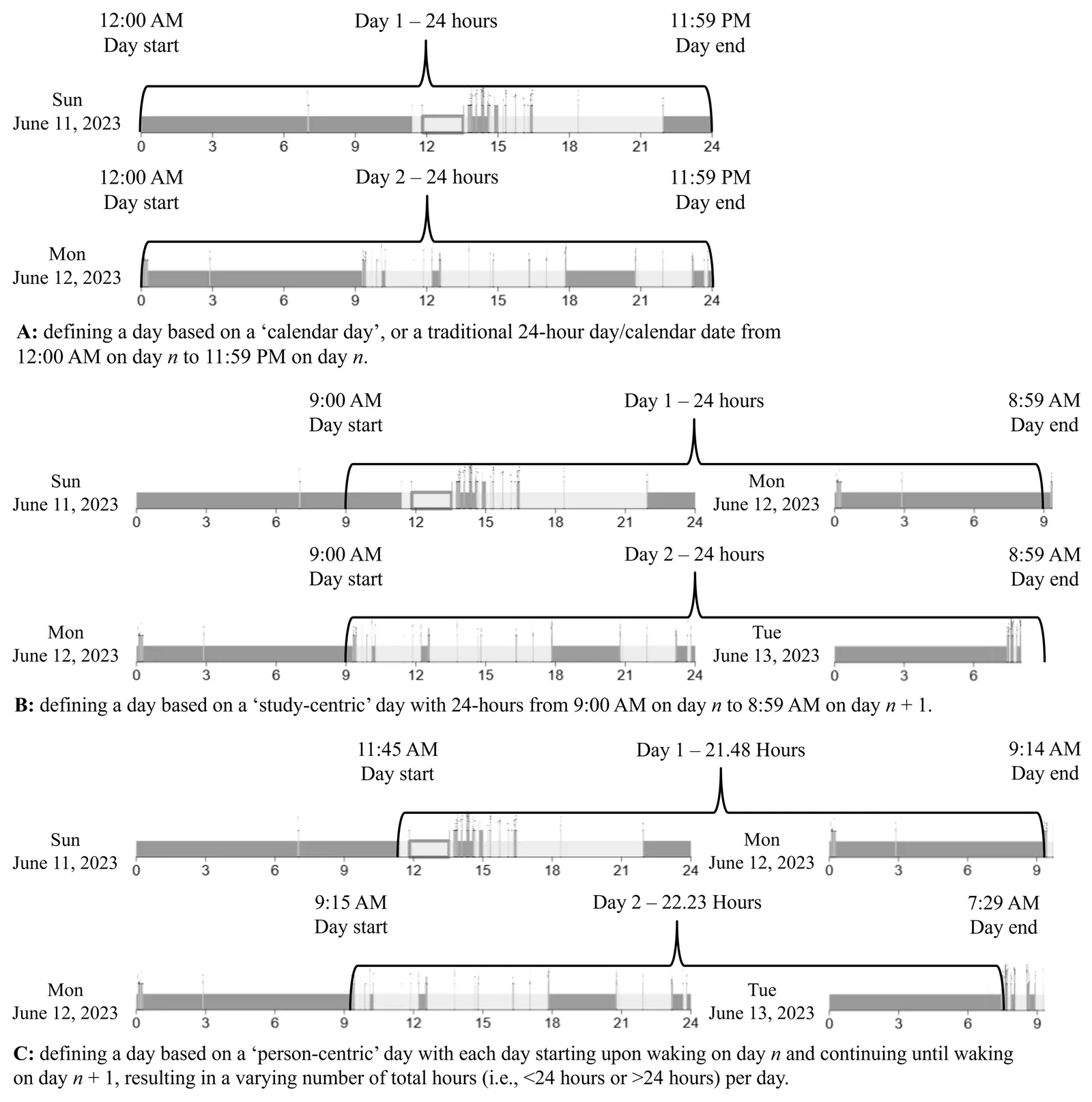
Decision 8: Scenarios for how to define a ”day” might be defined for a given study. (A) Defining a day based on “calendar day,” or a traditional 24-hour day/calendar date from midnight on day *n* to 11:59 PM on day *n*. (B) Defining a day based on a “study-centric” day with 24 hours from 9 AM on day *n* to 8:59 AM on day *n*+1. (C) Defining a day based on a “person-centric” day with each day starting upon waking on day *n* and continuing until waking on day *n*+1, resulting in a varying number of total hours (ie, <24 h or >24 h) per day.

After selecting the number and types of days to assess, the next decision is determining how a “day” is defined. For example, if a researcher is examining participants’ daytime behaviors, such as activities during work or school [48,89,100] or studying discrete events during the day, such as physical activity or drinking events [46,49,87], then defining the day based on a calendar day, which reflects the traditional 24-hour day or calendar date (eg, starting at midnight and ending at 11:59 PM) is appropriate (Figure 4). In general, a “calendar day” is appropriate when most participants go to bed before midnight or for studies examining daytime behaviors.

If a researcher is studying phenomena that are not contained within a calendar day, influence each other across the sleep-wake cycle, or have spillover effects across calendar days, then defining a “study-centric” day based on a key study feature, such as a morning EMA, is appropriate. For example, drinking events often occur after midnight and alcohol metabolism continues into the next calendar day. Using a study-centric day ensures these constructs are correctly attributed to the day when they practically occurred. For example, studies by Courtney and Russell [47] and Russell et al [51] used a 10 AM EMA as the daybreak when examining daily drinking intentions and outcomes.

**Figure 5.**
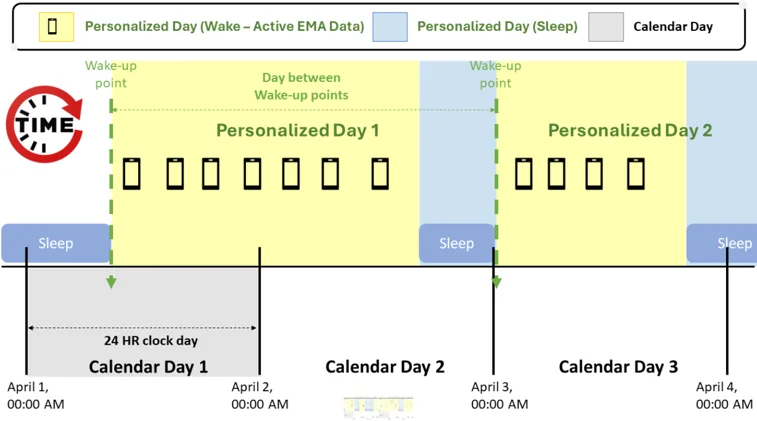
Decision 8: Example ecological momentary assessment sampling strategy when using a “person-centric” study day (reproduced from Wang et al [86]). EMA: ecological momentary assessments.

Finally, if a researcher is investigating variability in a person’s daily lived experiences or circadian rhythms, then a “person-centric” day based on an individual’s sleep-wake cycle (eg, not following a 24-h cycle) is appropriate. With a person-centric day, each day may differ between and/or within participants, and a “day” may include entire days or person-centric waking or sleeping periods. For example, Wang et al [86] were interested in within-person predictors of physical activity engagement and maintenance; therefore, they used person-centric days based on a participant’s sleep-wake cycle to examine these within-person processes (resulting in some “days” having more than 24 h and some less than 24 h; Figure 5). Researchers should decide a priori about how to define a “day” because it has important implications for sensor wear protocol, timing of measures, data processing, and results interpretation.

Application to research examples: In the first research example investigating affect and MVPA in children, the EMA sampling schedule was limited to 10 AM-8:30 PM; therefore, the design decision was to use a 24-hour calendar day. In the second research example investigating drinking intentions and behaviors, the design decision was to use a study-centric day. This was more appropriate than a 24-hour day because alcohol use often occurs after midnight and, even if a person is done drinking before midnight, alcohol metabolism continues throughout the sleeping period (ie, after midnight). By using a study-centric day, we avoid the problem of attributing sensor-detected alcohol use to the incorrect “lived” day, which would cause problems related to temporality and causality. The study-centric day was defined based on the start time of the morning daily diary, with the day running from 9 AM to 8:59 AM the following day. To further avoid issues of misattributing alcohol use to the incorrect data, it was also necessary to remove any residual alcohol detected after 9 AM when processing the sensor data.

## Decision 9: Determine Whether to Aggregate Data and, if Applicable, Which Assessments to Aggregate

After making the previous design decisions, researchers need to make data processing decisions related to aggregating assessments and temporally aligning variables based on whether the hypothesized associations are concurrent or lagged. These decisions are critical, because variation in decisions regarding which variables to include in analyses, which items to cluster with one another (eg, calculate average positive affect from several items), the handling of missing data, and the use of contemporaneous versus lagged variables all may impact final model results [101]. If there are multiple assessments per day (eg, EMAs), then the researcher needs to decide the timeframe and duration of time they are interested in studying (questions 32a and 32b). If a researcher is interested in all observations of the IV that occurred before DV, then they would aggregate all of those IV assessments during the specific time frame (taking the total, average, or variance value). If a researcher is interested in a duration of time shorter than 1 day, then they need to specify whether concurrent or lagged effects are of interest (questions 17‐19).

Data aggregation refers to combining multiple observations via summary measures, such as calculating average daily affect using multiple EMA responses within a day. There are two decisions underlying aggregation: (1) how to aggregate data (ie, which summary measures to use, such as mean, total, maximum, or variance), and (2) across which time frame (ie, measurement windows) to aggregate data (eg, hour or day). Both decisions can be informed by top-down (a priori) or bottom-up (data-driven) approaches. Top-down decisions may be preferable when guided by a specific research question. For example, if researchers are interested in the range of emotions children experience, they may aggregate using within-day variability (eg, variance) or the number of unique emotions reported, rather than mean levels of affect. Similarly, if researchers hypothesize that negative social interactions throughout the school day, but not after school, are most relevant for children’s emotion regulation, a top-down approach would support aggregating observations specifically within the school-day window. To facilitate implementation, Multimedia Appendix 2 includes example code for leading and lagging variables, data aggregation (ie, day-level mean, within-day variability, or number of unique emotions), and multilevel models. This code is intended as a starting point rather than a prescriptive template; researchers should carefully adapt all steps to their specific study design, research question, measurement structure, and analytic goals, particularly with respect to decisions about disaggregation (ie, within- vs between-person effects) and model specification (eg, inclusion of random slopes).

In contrast to top-down approaches, bottom-up, data-driven approaches (eg, machine learning methods) may be useful when there is limited theoretical guidance or when researchers aim to use empirical data for identifying optimal aggregation strategies. For example, researchers could compare multiple summary metrics (eg, mean, total, and variability) or time windows (eg, hourly vs daily) in terms of their predictive utility for an outcome, allowing the data to inform both the aggregation metric and time frame. Such an approach could reveal, for instance, that the total number of negative social interactions experienced in the morning has the largest impact on children’s emotion regulation.

Application to research examples: Research example 1 requires aggregating accelerometer data from the 30 minutes before and after the EMA prompts. Research example 2 requires aggregating alcohol sensor data across the study-centric day (9 AM-8:59 AM) so that only alcohol use after morning intentions is included in the models investigating day-level within-person associations.

## Limitations and Future Directions

Overall, this tutorial is meant to be a practical, step-by-step, decision-making tool to help beginning-to-intermediate ILD researchers design an ILD study. As such, it does not include advanced considerations needed for more complex research questions. The tutorial also does not address sophisticated ILD study designs that are needed to test time-varying effects (eg, within-person effects that vary across the day), time-varying moderating effects (eg, within-person effects that vary across contexts), multilevel mediation, idiographic effects, or N of 1 studies [102-105]. It also does not address research questions that examine within-person variability as a predictor, mediating, or moderating variable [106]. For example, a researcher may be interested in understanding how mood variability predicts changes in physical activity levels across childhood. The tutorial does not address some novel ILD measurement selection strategies, such as using computerized classification based on measurement error or mean and variability in previous EMA item responses to determine when and how often those items should be assessed [107,108]. Furthermore, the overall purpose of the tutorial is to inform the development of assessment protocols for observational studies, or as the evaluation component of intervention studies. It does not address considerations needed for the design and delivery of mobile or digital intervention strategies, such as when and how to provide intervention notifications that are part of microrandomized trials or JITAIs [14,15]. While the tutorial does provide some guidance on lagged versus leading observations and data aggregation, which is essential given that these choices can influence model outcomes and require transparency on the part of researchers [101], it does not address in depth the data processing and analysis needs inherent to ILD studies. For example, merging EMA data with sensor data based on time stamps and creating lagged or leading variables may require programming skills not typically taught in graduate training. Researchers may need to develop advanced data analytic skills or consult with data scientists and biostatisticians to ensure that data processing and analytic approaches are appropriately aligned with the a priori research question. The advent of AI systems provides another potential resource for researchers to use when encountering the data processing and aggregation challenges common to ILD. Furthermore, although the tutorial discusses selective data-processing decisions that have direct implications for study design (eg, temporal alignment and aggregation of observations), it does not provide detailed guidance on handling missing EMA responses, participant attrition, or incomplete sensor data. These issues are important considerations in ILD research and can have important implications for data quality and validity but were considered beyond the scope of the current design-focused tutorial [109]. Finally, the scope and emphasis of the tutorial are informed by the authors’ own research experiences and areas of applied work, which have influenced the selection of examples, populations, and design priorities. As a result, some ILD applications, research domains, or methodological perspectives may be underrepresented. While many of the principles discussed are broadly applicable, the tutorial primarily draws on examples from student and community populations and does not provide detailed guidance tailored to clinical or patient populations (eg, individuals with chronic conditions or older adults), for whom additional feasibility, burden, and adherence considerations may be especially important [41-44].

Further research in several key areas is needed to help researchers make better decisions about selecting measures and designing protocols for ILD studies. First, more comprehensive and systematic psychometric testing of daily diary and EMA items that compare different recall windows (eg, in the past hour vs 2 hours) and sampling densities (eg, 3 vs 10 prompts per day) can inform decisions about when and how often items should be assessed. Additionally, more thorough evaluations of the reliability and validity of EMA and sensor methods compared with established standards are needed. It would be helpful to develop an open-source repository that provides example items and detailed information about their timing, variability, and assessment burden within specific subgroups. Second, studies are needed to systematically test accessibility and usability of mobile, digital, and wearable assessment devices across vulnerable populations. For example, with older adult populations, the font on a smartwatch may be more difficult to see and vibrations on a sensor may be less detectable due to decreased skin sensitivity. Finally, future research directions include substantial opportunities to apply recent advancements in computational modeling, machine learning, and AI to predict the most opportune time to deliver the most useful type of ILD assessments to specific participants to reduce response burden and maximize the quantity and quality of the data.

## Conclusions

Advances in mobile technology and statistical methods have increased the accessibility and feasibility of collecting and analyzing ILD in health behavior research studies. However, a lack of readily available information and training on how to select measures and design the sampling protocol for ILD studies has hampered progress and the application of these methods. The tutorial provided here is intended to make it easier for researchers to implement rigorous ILD studies to enable scientific advancement and understanding of the nature of time-varying phenomena in health.

## Supplementary material

10.2196/81290 Multimedia Appendix 1 Decision-making tutorial for selecting intensive longitudinal data measures and designing a study using intensive longitudinal data.

10.2196/81290 Multimedia Appendix 2 R Markdown code file and .csv data file for example intensive longitudinal data coding and analysis.

## Abbreviations

DV: dependent variables
EMA: ecological momentary assessment
ESM: experience sampling methods
ILD: intensive longitudinal data
IV: independent variables
JITAI: just-in-time adaptive intervention
MVPA: moderate-to-vigorous physical activity
TIME: Temporal Influences on Movement and Exercise
