# Supplementary material for: Intensive Longitudinal Data Collection Methods in Health Research: Tutorial for Selecting Measures and Designing a Sampling Protocol
Source: JMIR Mhealth Uhealth. 2026 Sep 8;14:e81290. doi: 10.2196/81290 (PMC13552839; doi:10.2196/81290)
Supplement: Supplementary file 1 [file mhealth-v14-e81290-s001.docx]

| **Preliminary steps for determining whether an intensive longitudinal study design is appropriate for the research question** | | | | | | | |
| --- | --- | --- | --- | --- | --- | --- | --- |
| 1. What is your research question? | | | | | |  | |
| **DECISION 1: Determine whether the independent variable and dependent variable change over time within people.** | | | | | | | |
|  | | | | | | **Design Recommendations** | |
| 2. What is your primary dependent variable? ______________________________ | | | | | |  | |
| a. What is the specific definition you are using for your dependent variable? ______________________________ | | | | | |  | |
| b. Do you expect your dependent variable to change over time within people? Yes No | | | | | | ‘Yes’ 🡪 Suitable for ILD design  ‘No’ 🡪 Only suitable for ILD design if IV changes over time ^a^ | |
| 3. What is your primary independent variable? ______________________________ | | | | | |  | |
| a. What is the specific definition you are using for your independent variable? ______________________________ | | | | | |  | |
| b. Do you expect your independent variable to change over time within people? Yes No | | | | | | Yes’ 🡪 Suitable for ILD design  ‘No’ 🡪 Only suitable for ILD design if DV changes over time | |
| **If ‘Yes’ to #2 and/or #3, then proceed to the steps in the decision-making tutorial.**  **Below is a list of questions related to types of measurement, sampling strategies, etc. Provide answers to each question. When applicable, there are separate columns for providing answers for the primary dependent and independent variables. You may wish to go back and revise your research question as you answer these sections.** | | | | | | | |
| **DECISION 2: Identify the timescale of within-person changes.** | | | | | | | |
|  | | **Dependent Variable** | **Independent Variable** | | | **Design Recommendations** | |
| 4. What is the timescale across which the construct changes meaningfully within people? | | Seconds  Minutes  Hours  Days  Weeks  Months  Years  Doesn’t change | Seconds  Minutes  Hours  Days  Weeks  Months  Years  Doesn’t change | | | ‘Seconds’, ‘Minutes’🡪 Use Sensors  ‘Hours’ 🡪 Use sensors or EMA  ‘Days’ 🡪 Use Daily Diary  ‘Weeks, Months, Years, Doesn’t change’ 🡪 Do not use ILD | |
|  | |  |  | | |  | |
| **DECISION 3: Determine whether there are valid and reliable sensor-based, EMA, or daily dairy measures.** | | | | | | | |
|  | | **Dependent Variable** | **Independent Variable** | | | **Design Recommendations** | |
| 5. Is there a valid and reliable sensor-based measure (i.e., device that generates objective data) for the construct? | | Yes No | Yes No | | | ‘Yes’ 🡪 See #5a–5d  ‘No’ 🡪 Use EMA or Daily Diary | |
| If ‘Yes’ to #5: | |  |  | | | Assuming #5 is ‘Yes’: | |
| a. Does using the sensor result in *participant reactivity* to sufficient degree that it could alter study results? | | Yes No | Yes No | | | ‘No’ 🡪 Use Sensor  ‘Yes’ 🡪 Reduce participant access to sensor data | |
| b. Is the sensor cost-effective? | | Yes No | Yes No | | | ‘Yes’ 🡪 Use Sensor  ‘No’ 🡪 Use EMA or Daily Diary | |
| c. Are there resources (e.g., personnel, expertise, software) available to support using the sensor? | | Yes No | Yes No | | | ‘Yes’ 🡪 Use Sensor  ‘No’ 🡪 Use EMA or Daily Diary | |
| d. Does using impose too much *participant burden*? | | Yes No | Yes No | | | ‘Yes’ 🡪 Use EMA/Daily Diary or less frequent assessments  ‘No’ 🡪 Use Sensor | |
| If considering EMA: | |  |  | | |  | |
| 6. Is there an acceptable or widely used ecological momentary assessment (EMA) based measure for the construct? | | Yes No | Yes No | | | ‘Yes’ 🡪 See #6a–6d  ‘No’ 🡪 Modify between-person measure or, if timescale of change permits, use Sensor or Daily Diary | |
| If ‘Yes’ to #6: | |  |  | | | Assuming #6 is ‘Yes’: | |
| a. Does answering EMAs result in *participant reactivity* to a sufficient degree that it could alter study outcomes? | | Yes No | Yes No | | | ‘No’ 🡪 Use EMA  ‘Yes’ 🡪 Use Sensor or Daily Diary | |
| b. Does EMA significantly interrupt a *naturalistic process* that the participant is experiencing and/or change their response to that process? | | Yes No | Yes No | | | ‘No’ 🡪 Use EMA  ‘Yes’ 🡪 Use Sensor or Daily Diary | |
| c. Does the *naturalistic process* the participant is experiencing impact their ability to provide valid and/or reliable EMA responses? | | Yes No | Yes No | | | ‘No’ 🡪 Use EMA  ‘Yes’ 🡪 Use Sensor or Daily Diary | |
| d. Is EMA too costly for the researcher due to software, hardware, or research personnel costs, etc.? | | Yes No | Yes No | | | ‘Yes’ 🡪 Use Sensor or Daily Diary  ‘No’ 🡪 Use EMA | |
| If considering Dairy Diary: | |  |  | | |  | |
| 7. Is there a valid and reliable daily diary survey measure for the construct? | | Yes No | Yes No | | | ‘Yes’ 🡪 See #7a  ‘No’ 🡪 ILD may not be a suitable design choice | |
| If ‘Yes’ to #7 | |  |  | | | Assuming #7 is ‘Yes’: | |
| a. Are daily diary measures of the construct likely to be impacted by *expectancy effects*? | | Yes No | Yes No | | | ‘Yes’ 🡪 Randomize time of daily diary if possible  ‘No’ 🡪 Daily Diary | |
| **DECISION 4: Determine timing and frequency of sampling (*EMA studies only*)** | | | | | | | |
|  | | **Dependent Variable** | **Independent Variable** | | | **Design Recommendations** | |
| 8. Does the construct occur at the approximately the same times each day? | | Yes No | Yes No | | | ‘Yes’ 🡪 Interval-contingent EMA design (at times the construct occurs)  ‘No’ 🡪 See #8a | |
| If “No” to #8  a. Are you interested in measuring the construct at a particular time each  day? | | Yes No | Yes No | | | ‘Yes’ 🡪 Interval-contingent EMA design (at times the construct occurs)  ‘No’ 🡪 Signal-contingent or event-contingent EMA design | |
| 9. Are reports of the construct likely to be impacted by *expectancy effects*? | | Yes No | Yes No | | | ‘Yes’ 🡪 Signal-contingent EMA design  ‘No’ 🡪 Interval-contingent EMA design | |
| 10. Does the construct only occur during specific behaviors or events or in specific contexts or locations of interest? Or, are you only interested in the construct when it occurs during specific behaviors or events, or in specific contexts or locations of interest? | | Yes No | Yes No | | | ‘Yes’ 🡪 Event-contingent EMA design  ‘No’ 🡪 Signal or interval-contingent EMA design | |
| 11. If signal-contingent: How frequently do your constructs change across the day? | | Every hour  Every 2 hours  Every 3 hours  Every 4 hours  Every > 4 hours | Every hour  Every 2 hours  Every 3 hours  Every 4 hours  Every > 4 hours | | | Every hour🡪 Sample up to 16 times per day  Every 2 hours🡪 Sample up to 8 times per day  Every 3 hours🡪 Sample up to 5 times per day  Every 4 hours🡪 Sample up to 4 times per day  Every > 4 hours🡪 Sample 2-3 time per day | |
| 12. How long after it occurs or is experienced can a person reasonably recall the construct? | | Less than 15 min  Up to 1 hour  Up to 2 hours  Up to 4 hours | Less than 15 min  Up to 1 hour  Up to 2 hours  Up to 4 hours | | | ‘Less than 15 min’ 🡪 EMA recall period is “right now” or “in the past 15 min”  ‘Up to 1 hour’ 🡪EMA recall period is “in the past hour”  ‘Up to 2 hours’ 🡪EMA recall period is “in the past 2 hours”  ‘Up to 4 hours’ 🡪 EMA recall period is “in the past 4 hours”  **Note: Ensure that the recall period is not longer than the sampling frequency specified in #8.** | |
| **DECISION 5: Select sampling windows and time(s) of day when assessments occur (*Sensor and EMA studies only*)** | | | | | | | |
|  | | | | | **Design Recommendations** | | |
| 13. What is the overall sampling window of interest within each day? | | 24 hours  Sleep time only  Awake time only  Work time only  School time only  Other:_________ | | | ’24 hours’ 🡪 Continuous sampling via sensors &/or EMA  ‘Sleep time only’ 🡪 Sensor wear during sleep  ‘Awake time only’ 🡪 Sensor wear &/or EMAs while awake  ‘Work time only’ 🡪 Sensor wear &/or EMAs while at work  ‘School time only’ 🡪 Sensor wear &/or EMAs while at school | | |
| If “24 hours” or “Awake time only” for #13 and “Yes’s” for #6 (If not, skip)   14. Do participants have similar or different wake and sleep times from each other? | | Relatively similar  Very different | | | ‘Relatively similar’ 🡪 Use the same EMA daily sampling windows for all participants  ‘Very different’ 🡪 Create personalized EMA daily sampling windows | | |
| If ‘Very different’ for #14 (If not, skip)   15. Do participants have similar or different wake and sleep times each day? | | Relatively similar  Very different | | | ‘Relatively similar’ 🡪 Use the same EMA daily sampling windows for each day for each person  ‘Very different’ 🡪 Create different EMA daily sampling windows for each day for each person | | |
| **DECISION 6: Specify the temporal and causal associations of interest (*All ILD types*)** | | | | | | | |
|  | | | | | **Design Recommendations** | | |
| 16. Which type of association are you interested in testing? | | Whether within-person changes in the IV are associated with within-person changes in the DV  Whether between-person differences in the IV are associated with within-person changes in the DV  Whether between person-differences in the IV are associated with between-person differences in the DV | | | First answer🡪 Should be powered to detect within-person effects  Second answer🡪 Should be powered to detect between-person effects  Third answer🡪 Should be powered to detect between-person effects | | |
| If first answer choice selected for #16: (If not, skip)   17. Does your research question ask whether the independent variable is associated with the dependent variable at the same point (i.e., co-occurring) or later point (i.e., lagged) in time? | | Same point in time  Later point in time | | | ‘Same point in time’ 🡪 Ensure the IV and DV are sampled at same time and linked through date/time-stamps  ‘Later point in time’ 🡪 Ensure IV and DV can linked through date/time-stamps | | |
| 18. If same point in time for #17: What is the duration of that point in time? | | During the same <1-hour period  During the same <2-hour period  During the same <-4-hourperiod  During the same day-long period | | | If using EMA for the IV and/or DV🡪 Ensure that the EMA recall periods for the IV and DV (e.g., “right now,” “in the past hour,” “in the past 2 hours,” etc.) match the duration of interest  If using EMA for the IV and DV🡪 Ensure the EMAs for the IV and DV uses the same sampling frequency, recall period, and prompt times  Note: If the duration for #15 is shorter than the EMA sampling frequency and/or EMA recall period, then the latter two need to be shortened accordingly. | | |
| 19. If later point in time for #17: How much later in time? | | _____ minutes later  _____hours later  ______ days later | | | If using EMA for the IV and/or DV🡪 If the length of time for #15 is shorter than the EMA sampling frequency and/or EMA recall period, then the latter two need to be shortened accordingly. | | |
| **DECISION 7: Select number and type of days to assess** | | | | | | | |
|  | |  | | | **Design Recommendations** | | |
| 20. How many days of measurement are needed to ensure adequate representation or reliability of your constructs? | | ______ days | | | If Daily Diary only🡪 Consider whether the total number of days will be sufficient to detect a statistically-significant within-person association (at least 30 days per person is a good starting point) | | |
| 21. Are both weekend days and weekdays needed? | | Weekend days only  Weekdays only  Weekend days and weekdays | | | Weekend days only🡪Consider sampling across multiple weekends  Weekdays only🡪 Skip sampling on weekend days  Weekend days and weekdays🡪 Sample across weekend days and weekdays | | |
| 22. If EMA: How many observations per person will you assess in total? | | Multiply: EMA sampling frequency per day (in #8, #10, or #11 x EMA days of assessment (in #20) | | | Consider whether the total number of observations per person will be sufficient to detect a statistically-significant within-person association. (at least 30 per person is a good starting point)  Note: If more days of observation are needed, there should be fewer observations collected per day. If fewer days are needed, there can be more observations per day. | | |
| 23. If EMA: Is collecting EMA of the construct too burdensome for the participant due to EMA sampling frequency (from #11) or number of days of assessment (from #20)? | | Yes No | | | Consider sampling on random days with days off in between or splitting into a few separate, shorter sampling bursts of continuous days | | |
| 24. If Daily Diary: Are daily diary measures too burdensome for participants due to EMA sampling frequency (from #11 or number of days of assessment (from #20)? | | Yes No | | | Consider sampling on random days with days off in between or splitting into a few separate, shorter sampling bursts of continuous days | | |
| 25. Are you interested in whether the strength of the within-person associations change over a longer period of time? | | No  Yes, over weeks  Yes, over months  Yes, over years | | | No🡪 Use a single burst design  Yes, over weeks🡪 Use a multiple measurement burst design with bursts separated by weeks  Yes, over months🡪 Use a multiple measurement burst design with bursts separated by months  Yes, over years🡪 Use a multiple measurement burst design with bursts separated by years | | |
| **DECISION 8: Define what a ‘day’ means in the study** | | | | | | | |
|  | | | | | **Data Processing Recommendations** | | |
| 26. If interested in studying a 24-hour day or calendar date: Which parts of the 24-hour day or calendar date are you interested in studying? | Waking and Sleeping Behaviors  Waking Behaviors Only  Sleeping Behaviors Only | | | | ‘Waking and Sleeping Behaviors’ 🡪 See #27  ‘Waking Behaviors’ 🡪 Create custom wake period using sensor or self-reported bed and wake times  ‘Sleeping Behaviors’ 🡪 Create custom sleep period using sensor or self-reported bed and wake times | | |
| 27. If ‘Waking and Sleeping Behaviors’ for #26: Do the majority of your participants go to sleep before 12am on most days? | Yes No | | | | ‘Yes’ 🡪 Use a ‘calendar day’ with 12 AM as the day break  ‘No’ 🡪 Use a ‘study-centric’ day, with the mid-point of longest sleep period as day break or a pre-selected time (such as 4AM) during which the majority of participants are asleep as the day break or use the start of the daily study protocol as the day break | | |
| 28. Are you interested in studying a person’s daily lived experiences or circadian rhythms? | Yes No | | | | ‘Yes’ 🡪 See #29  ‘No’ 🡪 See other questions | | |
| 29. If ‘Yes’ for 28: Which parts of a person’s daily lived experiences are you interested in studying? | Waking and Sleeping Behaviors  Waking Behaviors Only  Sleeping Behaviors Only | | | | ‘Waking and Sleeping Behaviors’ 🡪 Create custom ‘person-centric’ day using sensor or self-reported bed and wake times (day lengths will vary)  ‘Waking Behaviors’ 🡪 Create custom wake period using sensor or self-reported bed and wake times  ‘Sleeping Behaviors’ 🡪 Create custom sleep period using sensor or self-reported bed and wake times | | |
| **DECISION 9: Determine whether to aggregate data and, if applicable, which assessments to aggregate** | | | | | | | |
|  | **Dependent Variable** | | | **Independent Variable** | | | **Data Processing Recommendations** |
| 30. How many assessments per person are you using to characterize one observation of the unit of interest (e.g., hour, half-day, day) for your DV and IV? | Single Assessment  Multiple Assessments | | | Single Assessment  Multiple Assessments | | | ‘Single Assessment’ 🡪 See #31  ‘Multiple Assessments’ 🡪 See #32 |
| 31. If ‘Single assessment’ for #30: |  | | |  | | |  |
| a. What timeframe is of interest? | Waking & Sleeping  Waking Only  Sleeping Only | | | Waking & Sleeping  Waking Only  Sleeping Only | | | See #31b |
| b. What is the timing of the observation you are interested in studying? (This should relate to responses to #17, #18, & #19) | Concurrent with IV  Prior to IV | | | Concurrent with DV  After DV | | | ‘Concurrent’ 🡪 Use single assessments when they occur concurrently  ‘Prior to IV’/’After DV’🡪 See #31c |
| c. What is the time lag between the observation and the IV/DV? | __ Minutes before IV  __ Hours before IV | | | __ Minutes after DV  __ Hours after DV | | | ‘Minutes’ before/after 🡪 Use the single assessment that occurs during this specific ‘minute’ time lag & during the timeframe from #31a  ‘Hours before/after 🡪 Use the single assessment that occurs during this specific ‘hour’ time lag & during the timeframe from #31a |
| 32. If ‘Multiple Assessments’ for #30: |  | | |  | | |  |
| a. What timeframe is of interest? | Waking & Sleeping  Waking Only  Sleeping Only | | | Waking & Sleeping  Waking Only  Sleeping Only | | | See #32b |
| b. What is the duration of time across which are you interested in studying? (This should relate to responses to #17, #18, & #19) | Entire time before IV  __ Minutes before IV  __ Hours before IV | | | Entire time after DV  __ Minutes after DV  __ Hours after DV | | | ‘All observations’ 🡪 Aggregate all assessments across timeframe specified in #32a  ‘Minutes’ before/after 🡪 Aggregate all assessments across this duration of minutes & across timeframe from #32a  ‘Hours’ before/after 🡪 Aggregate all assessments across this duration of hours & across timeframe from #32a |
| ^a^ Although there are circumstances in which ILD may be used when the independent variable changes over time but the dependent variable does not, this decision-making tutorial is based on research questions in which the dependent variable changes over time, as there are different methodological and statistical considerations required when the DV does not change over time. | | | | | | | |
